# Supplementary material for: Loss of the Novel Myelin Protein CMTM5 in Multiple Sclerosis Lesions and Its Involvement in Oligodendroglial Stress Responses
Source: Cells. 2023 Aug 17;12(16):2085. doi: 10.3390/cells12162085 (PMC10453064; doi:10.3390/cells12162085)
Supplement: Supplementary file 1 [file cells-12-02085-s001.zip › cells-2533818-supplementary.pdf]

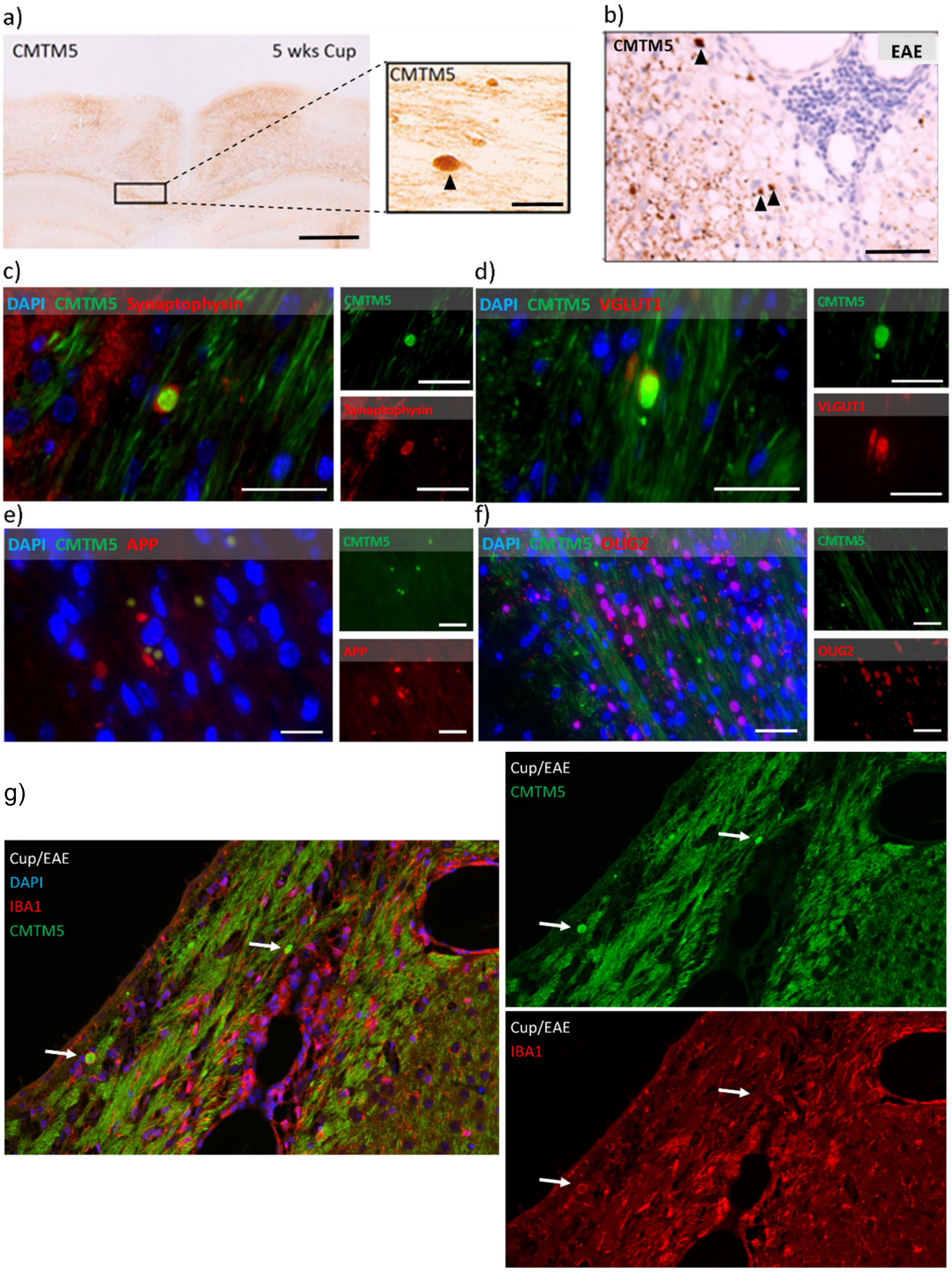

Figure S1. Colocalization of CMTM5-positive spheroids with markers for axonal damage. Representative images of anti-CMTM5 labellings showing its spheroid morhopology (arrowheads) in both the cuprizone demyelination model and **(a)** inflammatory demyelination EAE model **(b)**. Representative immunofluorescence double-labelled sections for anti-CMTM5 (green) and markers for axonal damage such as **(c)** anti-Synaptophysin (red), **(d)** anti-VGLUT1 (red), **(e)** anti-APP (red) after 5 weeks of cuprizone-intoxication. **(f)** CMTM5 was not colocalized with oligodendrocyte-lineage marker OLIG2. **(g)** CMTM5-positive spheroids were located within some IBA1-positive microglia/macrophages (arrows). Arrowhead indicates the perivascular cuffs where inflammatory infiltrates are observed in the murine forebrain using the combinatory CupEAE model [43].

Counterstaining was performed with DAPI (blue) to visualize cell nuclei. Scale bars: (a) = 200 µm, insert (a) = 10 µm, (b) = 30 µm. (c-d) = 50 µm. (e-f) = 25 µm. (g) = 40 µm.

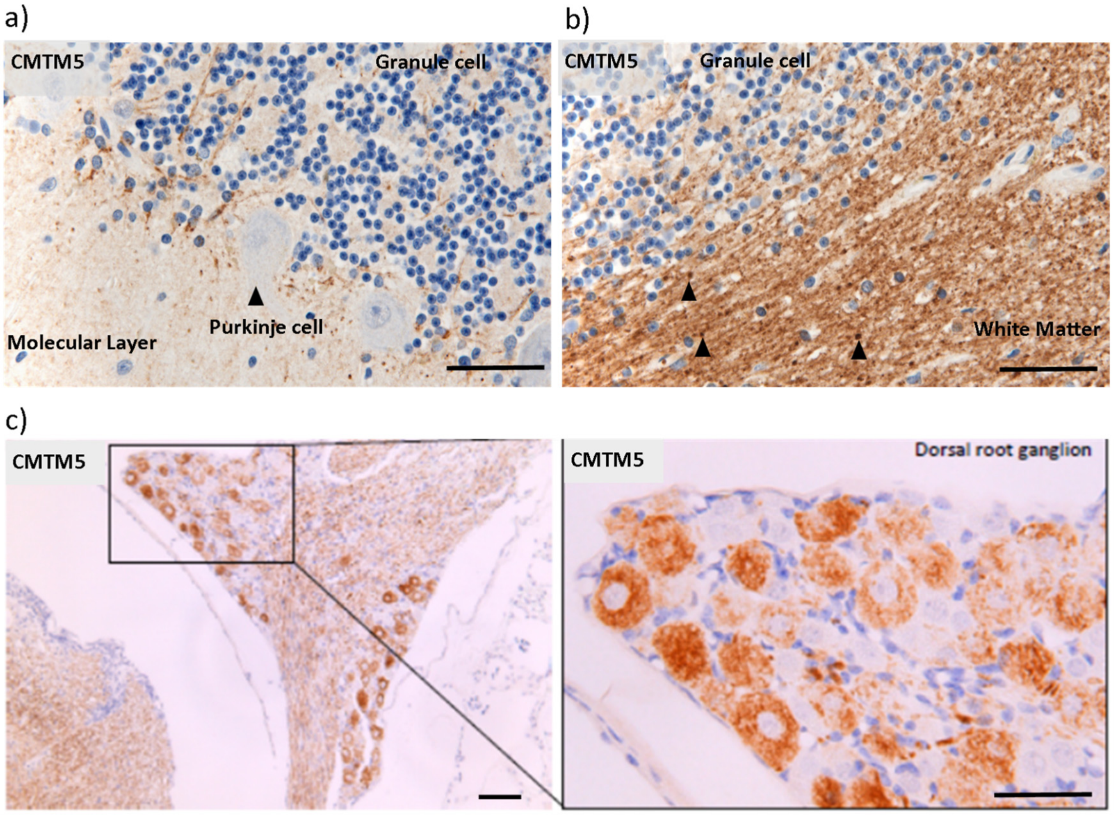

Figure S2. Representative images of anti-CMTM5 labellings resembling the morphology of myelin sheaths. **(a)** Purkinje cell layer of human cerebellum (arrowhead). Note CMTM5-positive fiber tracts are sparsely distributed surrounding Purkinje cells. **(b)** CMTM5 is widely expressed in the white matter of the human cerebellum. Arrowheads indicate CMTM5-positive spheroids. **(c)** Cytoplasmic CMTM5 immunoreactivity in dorsal root ganglion cells of murine spinal cord. Hematoxilin counterstaining was performed to visualize cell nuclei. Scale bars: (a-c) = 50 µm, insert (c) = 30 µm.

Table S1. Scoring results of experimental cohort "EAE". Daily scoring of EAE mice. The severity of EAE devlopment was scored as follows [6]: 1: the entire tail falls over the observer's finger when the mouse is picked up by the base of the tail; 2: the legs are not spread but are held close together when the mouse is picked up by the base of the tail, and the mice show a clearly visible wobbly gait; 3: the tail is flaccid and the mice show complete paralysis of the hind legs (a score of 3.5 is assigned if the mouse is unable to raise itself when placed on its side); 4: the tail is flaccid and the mice show complete paralysis of the hind legs and partial paralysis of the front legs, and the mouse barely moves in the cage but appears to be awake and eating; 5: the mouse is euthanized due to severe paralysis.

Days post immunization	EAE mouse#1	EAE mouse#2	EAE mouse#3	EAE mouse#4
0	0	0	0	0
1	0	0	0	0
2	0	0	0	0
3	0	0	0	0
4	0	0	0	0

5	0	0	0	0
6	0	0	0	0
7	0	0	0	0
8	0	0	0	0
9	0	0	0	0
10	0	0	1	0
11	0	0	2	1.5
12	1.5	0	3	2
13	3	1.5	2.5	2
14	2.5	2	2.5	2.5
15	2.5	2	2.5	2.5
16	2.5	2.5	2	2.5
17	4	3	3	2.5
